# Electrospun Shape Memory Polymer Micro-/Nanofibers and Tailoring Their Roles for Biomedical Applications

**DOI:** 10.3390/nano11040933

**Published:** 2021-04-06

**Authors:** Mohadeseh Zare, Pooya Davoodi, Seeram Ramakrishna

**Affiliations:** 1 School of Metallurgy and Materials, University of Birmingham, Birmingham B15 2TT, UK; m.zare@bham.ac.uk; 2 Department of Mechanical Engineering, Center for Nanofibers and Nanotechnology, National University of Singapore, Singapore 119260, Singapore; 3 School of Pharmacy and Bioengineering, Hornbeam Building, Keele University, Staffordshire ST5 5BG, UK; p.davoodi@keele.ac.uk; 4 Guy Hilton Research Centre, Institute of Science and Technology in Medicine, Keele University, Staffordshire ST4 7QB, UK

**Keywords:** shape memory polymers, electrospinning, molecular architecture, micro-/nanostructures, actuation methods, biomedical applications

## Abstract

Shape memory polymers (SMPs) as a relatively new class of smart materials have gained increasing attention in academic research and industrial developments (e.g., biomedical engineering, aerospace, robotics, automotive industries, and smart textiles). SMPs can switch their shape, stiffness, size, and structure upon being exposed to external stimuli. Electrospinning technique can endow SMPs with micro-/nanocharacteristics for enhanced performance in biomedical applications. Dynamically changing micro-/nanofibrous structures have been widely investigated to emulate the dynamical features of the ECM and regulate cell behaviors. Structures such as core-shell fibers, developed by coaxial electrospinning, have also gained potential applications as drug carriers and artificial blood vessels. The clinical applications of micro-/nanostructured SMP fibers include tissue regeneration, regulating cell behavior, cell growth templates, and wound healing. This review presents the molecular architecture of SMPs, the recent developments in electrospinning techniques for the fabrication of SMP micro-/nanofibers, the biomedical applications of SMPs as well as future perspectives for providing dynamic biomaterials structures.

## 1. Introduction

Stimuli-responsive materials have recently gained increasing attention in academic research and industrial developments as a relatively new class of materials that switch between a temporary and a permanent shape in response to a specific stimulus, including heat, pH-change, electrical or magnetic field, ultrasonic waves, and light. The main feature of shape memory polymers (SMPs) is to memorize the original shape of the structure before being exposed to a trigger and then reform after deformation (in the absence of the trigger) without any mechanical work. To achieve shape memory effects, a chemical architecture should be designed based on molecular switching segments and net-points [1,2]. Recent advances in SMPs have demonstrated a wide range of potential applications as their chemical structures and physical properties are highly adjustable to process needs [3,4]. SMPs studied as smart materials can be applied in various areas, including biomedical fields [5], fashionable textiles [6], aerospace [7], sensors [8], etc.

Nanotechnology, as a highly interdisciplinary research field, emphasizes the design of nanosized structures, including organic/inorganic nanoparticles, nanofibers, nanotubes, nanofoams, and nanopatterns. Electrospinning transformed SMPs’ scales down to the nanoscales, after the introduction of electrospun polyurethane-based SMP nanofibers in 2005 [9]. SMP nanofibers have drawn great consideration in technologies and science due to their distinctive properties, in addition to good shape recoveries, such as high surface area per volume unit, small diameters, high porosity, and low density. In virtue of such features, micro-/nanofibers have been extensively applied in the fields of filtration membranes, supercapacitors, and sensors, as well as biomedicine [8,10,11,12].

SMP micro-/nanofibers combine shape memory property and fiber characteristics which provide multifunctional structures. Interestingly, micro-/nanofibers present enhanced shape memory properties, compared to shape memory films [13]. Zhang et al. [14] discovered that SMP microfibrous membranes exhibited a considerably faster shape recovery rate compared to the SMP films of the same materials in the same conditions. That was attributed to the quicker heating/cooling rate of the fibers due to their larger specific surface areas. Moreover, SMP micro-/nanofibers can mimic the fibrillar features of ECMs and enable cell proliferation and migration as tissue engineering scaffolds [15]. Additionally, other structures, such as core-shell structures, developed by coaxial electrospinning have shown great potentials as drug carriers, artificial blood vessels, and wound dressings [16,17,18,19].

Besides, cell behaviors, including proliferation, differentiation, and alignment, are influenced by the dynamical behavior of ECM, signals transmitted by neighboring cells, growth factors, and cytokines [20,21,22]. The dynamic behavior of ECM can regulate cell functions and accelerate tissue regeneration [23]. By endowing SMPs with micro-/nanoscale features, the interaction between the microenvironment and cells can be properly tuned and controlled. For instance, researchers have recently developed micro-/nanofiber platforms and micropatterned structures with reversible interactions to regulate cell behaviors and tissue formation [24,25]. Moreover, the shape memory property allows the polymers to be implanted and fill irregular defects through minimally invasive surgery.

**Figure 1:**
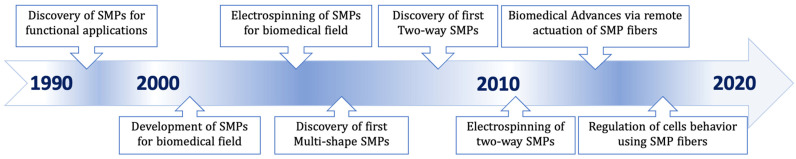
A timeline chart of the progress in shape memory polymers (SMPs) discoveries, electrospinning, and biomedical applications.

SMPs have been developed over the last three decades [26], as shown in Figure 1. The development of biodegradable SMPs [27], the multishape SMPs [28], two-way SMPs [29,30] and the electrospinning of SMPs for the biomedical applications [31] occurred in the 2000s. During the past decade, there has been a tremendous progress in biomedical advances using the remote actuation of SMP fibers [32] as well as the regulation of cells’ behavior through taking advantage of SMPs’ fibrous structures [33].

While general principles of SMPs, their material chemistry, and structures have been extensively reviewed previously [5,34,35], the current review mainly focuses on recent advances in the preparation of shape memory micro-/nanofibrous structures via electrospinning, and sheds light on their biomedical applications in minimally invasive surgery, drug delivery, bone repairs, vascular grafts, wound dressing, and cell regulation.

## 2. Architecture of SMPs

**Figure 2:**
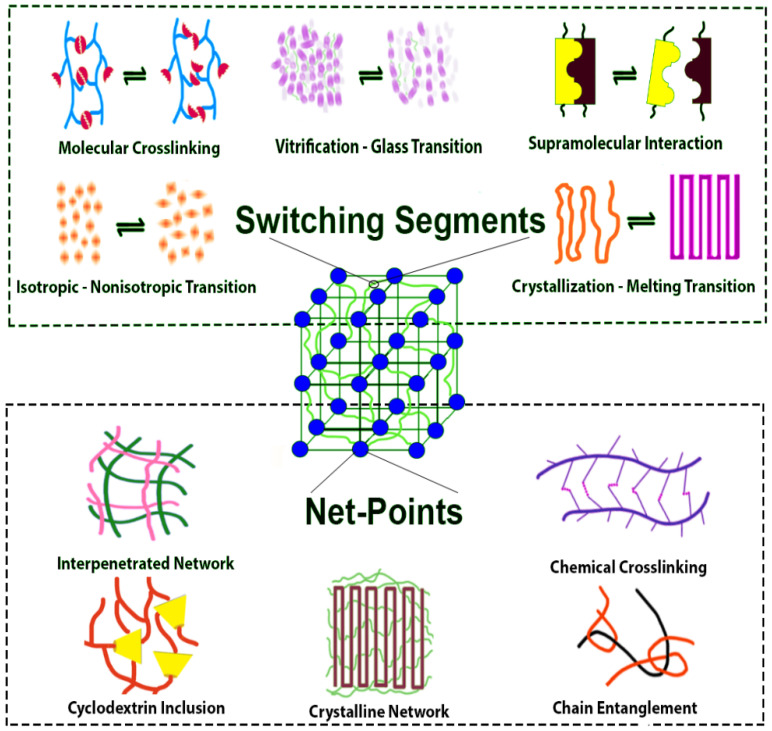
The overall schematic of the molecular architecture for SMPs consisting of switching segments and net-points.

SMPs are comprised of a permanent and a temporary shape resulted from a combination of the molecular chemistry and a programming procedure [36]. The required chemical architecture involves net-points and molecular switches with a sensitivity to a stimulus. In the same sense, SMPs can also be considered as copolymers with hard and soft segments acting as fixed and reversible phases, respectively. The fixed phase prevents the flowing of the polymer chains upon applying stress while the reversible phase experiences deformation and behaves as a molecular switch. This phase freezes the temporary shape during stimulation while it returns to the original shape when the stimulation is switched [8]. The molecular mechanisms of SMPs architecture illustrated in Figure 2 can be applied to any kinds of SMPs. In this scheme, SMPs are made up of both net-points and molecular switches. This fixed phase (i.e., net-points) can be achieved by the introduction of crystalline phases, chemical crosslinks, chain entanglements, interpenetrating networks, or interlocked supramolecular complexes. Switch parts are responsible for shape fixity and recovery upon applying an external stimulus. The crystalline, liquid crystalline, and amorphous phases, supramolecular entities, light-reversible couplings, and percolating cellulose-whisker networks have been introduced as the switch units in SMPs [36]. The reversible phase fixes the temporary shape through glass transition, crystallization, isotropic transition, supramolecular interactions, and reversible covalent or noncovalent bonds.

Shape memory Polyurethanes (SMPUs) are the most prominent type of SMPs for biomedical applications because of their biocompatibility, biodegradability, easily manipulated structure, and transition temperature near to body temperature [37]. SMPUs are segmented block copolymers with hard and soft separated segments. The soft switching segments are generally composed of long-chain diols such as polyester or polyether polyols, whereas the hard permanent segments are mainly formed by the reaction of short-chain diols including chain extenders with diisocyantes as physical crosslinkers [36,38].

Shape memory behavior can be caused by different states of molecular chains under various stimulations. In other words, the shape memory effect (SME) of such materials can be divided into three different categories of (i) one-way SME, (ii) multiple-SME, and (iii) two-way (reversible) behavior, depending on the different shape-recovery mechanisms described in the following sections.

## 3. Triple and Multiple SMPs

Triple SMPs possess two temporary states in addition to the permanent shape. The SMPs transform from the first to the second temporary shape by stimulating, and another stimulation makes the deformation back to the permanent shape. These types of SMPs are common among thermoresponsive SMPs and there are two main strategies to design them: (i) a broad thermal transition temperature (*T*_trans_) and (ii) multiphase designation, in which each phase proposes a separate transition [39]. However, the polymerization-induced phase separation can also be considered in making a multiphase morphology [40]. Mather et al. utilized this strategy to induce a triple SMP composite based on poly (ε-caprolactone) (PCL) and epoxy [41]. Block copolymers also provide a possibility to prepare a nanophase separated polymer morphology and consequently offer two separate thermal transitions needed for triple SMPs [42]. According to this strategy, Gao et al. prepared a gradient polymeric structure which caused a gradual shifting of the glass transition temperature (*T*_g_) and consequently a broad glass transition [43].

In addition, Li et al. could achieve a quintuple-SMP using a semi-interpenetrating network with extended crystalline and glass transition segments. This provided multigradient *T*_g_ and a melting temperature as transition temperatures for multistep shape changes in one shape memory cycle [44].

Polymer composites containing different phases can also be utilized individually to create multishape SMPs. For instance, an SMP epoxy matrix reinforced by PCL fibers induced two thermal transitions (i.e., *T*_g_ of the matrix and *T*_m_ of the PCL fiber) [45]. Zhang et al. could also electrospun Nafion, comprising perfluoro ether sulfonic acid side chains and polytetrafluorethylene backbone, via adding a small amount of poly (ethylene oxide) [46]. This electrospun SMP, showed a broad transition temperature and could be designed to memorize five shapes at different transition temperatures, providing potential applications for sensors, smart textiles, actuators, and artificial skins.

## 4. Reversible SMPs

The SME explained so far is a one-way SME that is missing the reversibility of the shape. It means that when the SMP recovers its permanent shape, another step is necessary to apply to reconstruct the temporary shape. A suitable shape memory material has to be able to switch between its shape reversibly several times without applying an external reshaping process. Therefore, the temporary shape should reform itself upon terminating the stimulus. This kind of SMPs is called reversible or two-way SMP [35]. Despite the increasing knowledge about designing SMPs, two-way SMMs are still scarce. To obtain such SMPs, an internal driving force for the reverse transformation is compulsory. The switching phases are based on reversible mechanisms which the most common ones will be discussed in the following sections.

### 4.1. Reversible Thermally Induced SMPs

**Figure 3:**
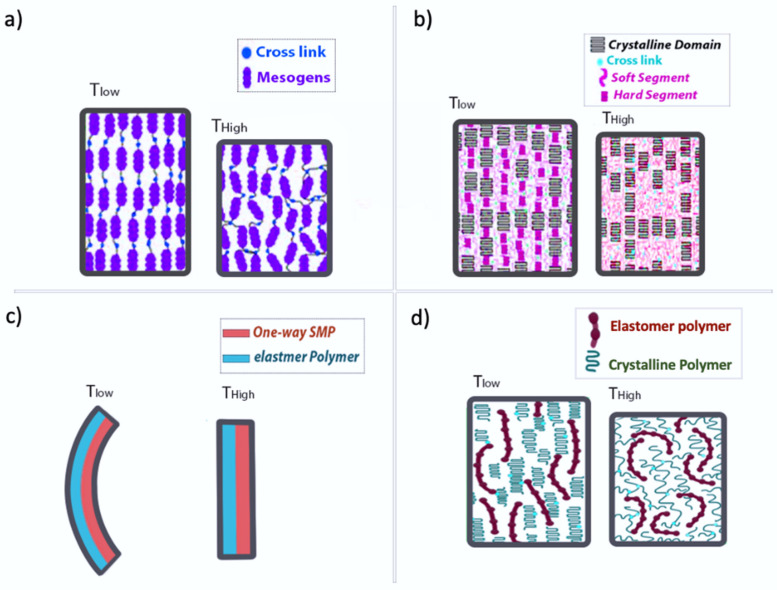
Schematic of the strategies for thermally induced two-way SMPs: (**a**) liquid crystalline elastomer; (**b**) semi crystalline network; (**c**) polymeric composite network, (**d**) interpenetrating network.

Thermally induced SMPs are the most widely studied type of SMPs [35]. A design strategy for reversible thermally induced SMPs is based on liquid crystalline elastomers (LCE) which undergo a transition between an isotropic and anisotropic phase (Figure 3a). In other words, two-way SMPs of LCEs result from the combination of the elastic characteristic of the network and the arrangement of mesogenic units [47]. For instance, Mather et al. [48] synthesized a two-way SMP based on a liquid crystalline monomer polymerized by acyclic diene metathesis and crosslinked.

Semicrystalline networks (SCN) have been considered as another strategy for reversible SMPs due to their simple chemistry and easy tailoring of the transformation temperatures. Mather et al. [30] reported a crosslinked poly (cyclooctene) as an SCN that undergoes a crystallization induced elongation during cooling across the melting temperature in the presence of constant tension (Figure 3b). The elongation was reversed and melting induced contraction appeared upon heating above the melting temperature.

Two layered polymeric networks formed with the aid of adhesives instead of by covalent linkages have been investigated as another strategy for thermally induced two-way SMPs (Figure 3c). These two polymer layers can be made of one-way SMPs as well as a layer of one-way SMP with an elastomer layer [29,49]. There are also other works for the fabrication of two-way shape memory composites without using SMP components. As an example, Tamagawa et al. fabricated a two-way SMP using two ordinary polymers without the shape memory property [50].

The most recent strategy for the preparation of thermally induced two-way SMPs is the use of the interpenetrating network (IPN) of the elastomeric and crystalline polymers introduced by Wu et al. [51]. IPN is made of a combination of at least two polymeric networks with a molecular interlacing within the matrix and no covalent bonds between the networks (Figure 3d) [52]. A variety of methods are used to synthesize the IPNs, but the photopolymerization of monomers or oligomers is the most common technique used to prepare IPN [51].

Thermoresponsive SMPs typically require a programming process in addition to the molecular architectures. The programming is based on a cyclical thermomechanical process which has been used and reported by several authors [51,53,54,55]. For instance, two-way PCL-based SMPs were created via combining the electrospinning method and sol–gel crosslinking reactions and were subsequently programmed for the shape memory behavior [54].

### 4.2. Reversible SMPs Based on Thermo- and Light-Activated Covalent Bonds

**Figure 4:**
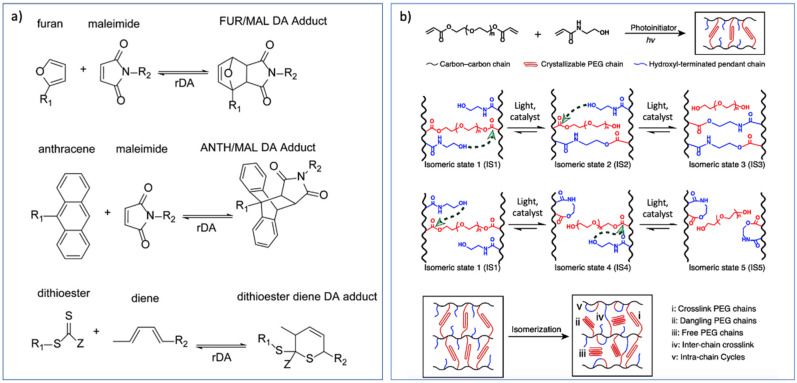
(**a**) Thermoreversible Diels–Alder adducts including Furan-maleimide, Anthracene-maleimide, and Dithioester diene (with permission from Wiley, Copyright 2016 [56]); (**b**) isomerization reaction via interchain and intrachain transesterification pathway (Reprinted with permission from ref. [74]. Copyright 2020 Nature).

The switching phases required for reversible SMPs can also form via reversible covalent bonds. Particularly, the Diels–Alder and Retro-Diels–Alder reactions are well-known for this purpose. The most common forms of thermoreversible Diels–Alder reactions include the furan-maleimide, anthracene-maleimide, and dithioester diene DA adducts as shown in Figure 4a [56]. Raquez et al. [57] and Alexandre et al. [58] synthesized four-arm star shaped PCL, functionalized with anthracene, maleimide, and furan moieties, and subsequently reactive extrusion created crosslinked networks. Besides the end groups functionalization with Diels–Alder reactions, Yoshie et al. synthesized a polymer using furan moieties in the polymer chains [59] crosslinked it with bismaleimides to achieve a SMP based on reversible Diels–Alder moieties [60]. Another reversible covalent interaction to design SMPs is disulfides. Rowan et al. synthesized a poly-disulfide, which could perform self-healing by light radiation and SME using thermal treatment [61].

Supramolecular interactions such as ionic interactions, hydrogen bonds, or metal-ligand interactions can also be utilized instead of reversible covalent bonds for designing SMPs. Due to easy adjusting of the strength of bindings and straightforward creation of multifunctional polymers, hydrogen bonds can be utilized for the preparation of SMPs [62]. Wang et al. synthesized a four-arm star shaped PCL with the hydrogen bonding and added four-arm polydioxanone with hydroxyl end groups. This system could show a shape memory behavior as a result of hydrogen bonds as well as an interpenetrating network [63].

In addition to hydrogen bonds, ionomers were also investigated as a supramolecular interaction. The most common approaches are sulfonated polymers neutralization with zinc salts [64] and ionic groups integration via carboxylic acids [65].

Besides the above-mentioned supramolecular interactions, metal–ligand interactions also provide reversibility for the design of SMPs. Rowan et al. prepared a permanent covalent network, created by a thiolene reaction with tetrafunctional thiol crosslinker, and a reversible phase, was subsequently achieved by complexing the ligand with europium ions [66].

Development in photoactivated covalent chemistry has increased the interest for the design of SMPs based on light-triggered covalent bonds SMPs for biomedical application because of their easier remote actuation compared to thermal actuation. The most general example for light activated SMPs is the photodimerization of cinnamic acid derivates as reversible elements introduced by Leindlein et al. [67]. Nagata et al. prepared the polyester, containing cinnamic acid in the main chain, which could stimulate the prepared gel upon exposure to ultraviolet (UV) light [68,69].

Coumarins can undergo photocrosslinking and reversible transitions based on cycloaddition reactions for the designation of photoinduced SMPs. He et al., further improved this idea via combining the dimerization of coumarin with hydrogen bonds to obtain a photoswitchable structure [70].

Photoisomerization reactions can be also employed for the preparation of photo-induced SMPs. The most important example of these reactions is a cis–trans isomerization of azobenzene moieties upon light exposure [71]. In that case, White et al. synthesized an azobenzene-containing diacrylate, as a crosslinker which can recover the original shape after light illumination [72]. Besides the azobenzene as a photoisomerization, the spiropyran isomerization reaction can also be utilized. Liu et al. examined the shape recovery of spiropyran doped ethylene-vinyl-acetate copolymer based on the isomerization of spiropyran [73].

Moreover, Miao et al. used the concept of topological isomerizable network to design a light-triggered SMP based on polyethylene glycol diacrylate (PEGDA) offering ester bonds [74]. The ester bonds could undergo transesterification reaction with the pendent hydroxyls as shown in Figure 4b.

## 5. Stimulus-Responsive Methods

The stimulation methods play a leading role for the practical applications of SMPs where various triggers including light, heat, pH, moisture, water, and electrical and magnetic fields, and have been successfully utilized in research studies [36,47].

The most widespread and straightforward type of stimulation is the direct thermal activation of SMPs based on two thermal transitions: (i) the melting temperature and (ii) the glass transition temperature. The melting transition temperature can be utilized in chemically and physically crosslinked polymers as well as in semicrystalline networks. This type of SMPs presents (multi)block copolymers with a low melting phase, as a switching segment, and a high melting phase, as a permanent network. Similarly, the glass transition can be utilized in physically crosslinked thermoplastics as well as chemically crosslinked thermosets. Most melting-temperature-based (*T_m_*-Based) SMPs investigated are based on polyolefins, polyethers, and polyesters with a soft phase of low melting temperature, while their crystalline hard phase remains untouched at an elevated temperature [35]. In these cases, the switching temperature depends on the degree of branching as well as the crosslinking density.

The polymers typically demonstrate a glass transition below the room temperature. Thus, polymeric materials with a *T_g_* above 25 °C can be utilized as *T_g_*-based SMPs. In comparison to *T_m_*-based SMPs, *T_g_*-based SMPs show a slower shape recovery due to the broad glass transition. Consequently, *T_g_*-based SMPs are not ideal candidates for applications where a quick shape recovery process is required. However, the slow shape recovery process is attractive for biomedical applications [75].

By increasing the crosslinking density, higher glass transition temperatures and consequently, higher switching temperatures can be achieved. Recently, thermosetting shape memory cyanate polymers have been investigated with a glass transition temperature higher than 250 °C [76]. Therefore, these materials show high thermal stability and excellent shape recovery and shape recovery.

The direct thermal actuation of SMPs can somewhat limit the range of their applications due to the need to control the temperature using a heat source such as an oven or water bath. Nanotechnology has been assumed to solve this difficulty in obtaining various indirect thermal actuation. Adding nanoparticles into the SMPs matrix can create heat within the construct using a remote energy source. Indirect heating methods including applying magnetic, electrical fields, microwaves, UV, and NIR irradiations can meet the needs of many practical applications by offering remote controlling the temperature. The most common nanofillers used in different SMPs are Fe3O4, Au, Ag, Ni, CNTs, SiC, graphene oxide, and cellulose nanocrystals [10,77,78,79,80,81]. Molecular vibration in these systems plays the role of generating heat indirectly. For example, magnetic fillers have been widely employed for the heat activation of SMPs where the energy is induced by altering a magnetic field, and the concentration and size of the particles determine the magnitude of the energy conversion and the final temperature of the SMP [82,83].

In addition to the direct and indirect thermal stimuli described so far, there are other activation methods with the potential for future applications, including ultrasonic waves [84,85], mechanical pressure [86], and water- or solvent-induced activations based on chemical bindings and physical swelling [87,88,89]. The addition of organic salts can endow SMPs with water-induced shape recovery through the dissolution of water soluble salts and the formation of porosity within the SMP construct [90]. In another research paper, a moisture-responsive fibrous membrane was prepared by Dallmeyer et al. using electrospinning of Kraft lignin fractions to have a two-way SME [91].

Researchers have also designed SMP composites with multiple simultaneous activation mechanisms to precisely tune shape memory performances [92]. A recent study demonstrated a polystyrene-based SMP composite filled with CNT, Fe_3_O_4_, and pure polystyrene which could present a selective actuation in the exposure of an alternating magnetic field [93]. Such SMPs could be applied for applications requiring complex reconfigurable structures. This system exhibited not only a selective activation but also multiple shape changing functions with three distinct defined shapes.

## 6. SMPs with Biomimetic Micro-/Nanofibrous Structures

At the beginning of the development of SMPs, they were applied in balk forms which could narrow their field of applications [36,94]; but, with the development of nanotechnology, the requirements of many potential applications have been fulfilled. Nanofabrication techniques that have been used to achieve the necessary micro-/nanostructures include foaming, spin coating, electrospinning, 3D or 4D printing, and transfer printing. These methods have allowed SMPs to form nanofibers [95], porous films [86], micropatterns [96], micro or nanoparticles [97], and foams [98]. SMPs in these forms can extend their potential applications in biomedical fields.

Many types of natural and synthetic polymers can be utilized for synthesizing biomimetic SMPs. Natural polymers, such as alginate [99,100], chitosan [101,102], and collagen [103] have been applied for the fabrication of porous SMP scaffolds. Synthetic polymers, like PCL [32,104,105], poly (D, l-lactide) (PDLLA) [106,107,108], polyurethane (PU) [10,14,109], poly (lactic-co-glycolic acid) (PLGA) [110,111], epoxy polymers [112,113] and polyacrylates [114,115], can be usually crosslinked by chemical interactions or polymer chains with a high glass transition temperature (*T_g_*) to form permanent networks for SMPs. Polymer components with the melting temperature (*T_m_*) or low *T_g_* prepare the switching network of the SMPs. To improve the SMP properties and functionalize them for the biomedical applications, they can also be combined with drugs, and various nanoparticles such as hydroxyapatite (HAp) [107,116,117], multiwalled carbon nanotubes (MWNTs) [32,118], cellulose [119,120], and graphene oxide (GO) [10,79].

Although biomimetic SMPs have biocompatibility, for particular applications, such as absorbable scaffolds, biodegradability is also essential because biodegradation eliminates the second surgery for removing the construct after the treatment. However, the challenge is how to match the required time for degradation with the performance period of SMP materials. Therefore, biodegradable SMPs should be designed with a tunable degradation rate, maybe through the variation of the SMP compositions [121,122].

In addition, micro-/nanofibrous structures can endow the SMPs with flexibility, easy deformation, and controlled porosity and offer significant potential for cell growth applications [106]. Cells are typically cultured on static surfaces; recently, researchers have reported cell culturing on fibrous scaffolds programmed to alter their morphology during cell culture. For example, a polyurethane based SMP as a nanofibrous scaffold has been used to examine the hypothesis that the alignment of SMP fibers can regulate the morphology and behavior of the cells attached to the dynamic surface [33].

## 7. Development of Electrospinning Process for SMPs

Combining the SME with a micro-/nanofibrous structure endows the polymers with some distinctive properties for many applications [106]. SMPs with micro-/nanofibrous structures can be produced using electrospinning process [24], extrusion spinning [123], or melt spinning [124]. Among these methods, SMP fibers prepared by electrospinning can have various diameters in the micro-/nanoscale and homogenous morphologies through adjusting the operating parameters, the solution properties, and the environmental condition.

Electrospun fibers usually have characteristics of uniform diameter, round cross section, and smooth surface. SMPs can be electrospun into different fibrous morphologies, including nonwoven fibers [13], aligned fibers [125], core/shell fibers [126], and fibers with a functional particle filling [32] by making some changes to the conventional electrospinning equipment.

### 7.1. Conventional Electrospinning Process

Electrospinning technology has been introduced as the most efficient method to convert polymers into continuous fibers [127]. During electrospinning, the polymer solution droplets overcome the surface tension and create a conical shape under the electrostatic interaction. The electrostatic force stretches a jet flow and divides into ultrathin fibers which can be collected after the solvent evaporation [128,129]. The distance between the nozzle tip and collector, applied voltage, flow rate, and solution parameters such as viscosity and conductivity, have critical effects on electrospun fibers’ characteristics [130,131].

Due to the mentioned advantages, fibrous SMPs have been increasingly investigated [132,133,134,135]. Cha et al. electrospun a synthesized polyurethane-based SMP for the first time in 2005, which revealed a shape recovery of more than 80% [9]. Leng’s group could fabricate nonwoven SMP nanofibers via the electrospinning method from Nafion and poly (ethylene oxide) solutions with shape recovery ratios and shape fixity ratios of more than 90%. It was confirmed that SMP fibers were stable after the stretching recovery [134].

Hu et al. also fabricated SMPU fibers and found that after several cycles of thermomechanical programming the shape recovery was increased to 98% [12]. Zhang et al. also found that microfibers have quicker shape recovery than SMPU films when heated, which can be attributed to the high-specific surface area of fibrous SMPs. [14]. Recently, Lendlein’s group prepared a microscaled nonwoven scaffold by electrospinning of a copolymer, consisting of crystallizable poly-pentadecalactone as hard segments and PCL as switching segments, with potential applications in biomedicine [136].

To fulfill the requirements of various applications, the electrospinning parameters can be adjusted to fabricate various structures like aligned fibers, beaded fibers, ribbons, and porous fibers [24,33,137].

Yoo et al. [138] investigated the shape memory behavior and mechanical properties of a PCL-based SMP in the form of randomly oriented fibers. Zhou’s group [32] fabricated shape memory aligned fibers using a high-speed drum to collect the fibers. Tseng et al. [33] also used aligned fibers to investigated the cell behavior on membranes made of different oriented fibers [137]. Furthermore, the moisture-responsive Kraft lignin-based fibers prepared by electrospinning, were reported by Dallmeyer et al. [91] to have a two-way SME. In another study, porous water-responsive SMP, containing poly (ethylene glycol), PCL, and poly (dimethylsiloxane) were fabricated by electrospinning process which showed good shape fixity and shape recovery when immersed in water [139]. A conductive SMP based on poly (lactic acid) was prepared by combining electrospinning process with vapor polymerization. The poly (lactic acid)-based SMP was electrospun and then coated by conductive polypyrrole using the vapor polymerization to prepare a conductive SMP which can be utilized as sensors and actuators [140].

Moreover, the size of the pores in the SMP membranes prepared by electrospinning was investigated by Ahn et al. [141]. Following the temperature changes, the pore size can change. This phenomenon proved that the SMP fibers can form smart membranes to selectively separate particles via controlling the temperature.

### 7.2. Coaxial Electrospinning

While traditional electrospinning was limited to the polymers featuring good spinnability, the coaxial electrospinning method provided a simple way to use the polymers without spinnability in fibrous structure [142]. By using two or multiple nozzles in a concentric geometry, a coaxial electrospinning system can be set up and a core-sheath structure of the fibers can be achieved based on the formation of a charged compound jet of concentrically flowing solutions. After the preparation of the core-shell fibers, one can selectively dissolve core polymer in an appropriate solvent to obtain hollow fiber structures. As an example, Lendlein et al. [143] prepared SMP hollow fibers based on copolyetheresterurethae by coaxial electrospinning technique and poly (ethylene glycol) as the sacrificial core.

Zhang et al. applied coaxial electrospinning method to prepare a core/shell fibrous composite using epoxy as the core and PCL as the shell [126]. The obtained fibers exhibited excellent shape memory performance with enhanced mechanical properties for potential biomedical applications.

SMP fibers with core-shell structure and bead-on-string structure have also been fabricated by designing the spinneret containing two coaxial capillaries [19]. The core-shell nanofibrous SMP exhibited good antibacterial activity against Gram-negative and Gram-positive bacteria. The antibacterial mechanism was based on amino groups of shell materials and the high surface area of nanofibers. Therefore, the prepared core/shell nanofibers could be applied as antibacterial nanofibrous SMPs.

### 7.3. Electrospinning of SMPs with Functional Fillers

Besides preparing the SMPs’ nanofibrous structures, some functional materials, such as carbon nanotubes, graphene oxide [10], cellulose nanoparticles [119], HAps [116], epoxy resin [112,113], and magnetic particles [32,55] (Fe_3_O_4_, Au, Ni, etc.) can be incorporated in the fibers during electrospinning to endow the fibers with a multishape memory property, on-demand actuation using external stimulations, electrical conductivity, self-healing properties, and antimicrobial properties. As an example, Gong et al. reported the incorporation of Fe_3_O_4_ into the SMP nanofibers which were able to be triggered by the magnetic field, as a remote control [32]. Magnetic nanoparticles can absorb magnetic energy from a magnetic field applied and produce local heat which results in the thermal actuation and deformation of the SMP scaffold.

With the development of electrospinning of SMPs with diverse functional fillers, different stimuli have been achieved in recent studies [32,139,144]. As mentioned previously, among the various actuation methods the, direct and indirect thermal actuation of SMP micro-/nanofibers were the most commonly studied. For instance, Zhou et al. [32] fabricated PCL-based SMP nanofibers as the matrix and multiwalled carbon nanotubes coated with Fe_3_O_4_ as the reinforced filler, which could be triggered both by a magnetic field and hot water. Additionally, Sabzi et al. utilized dual electrospinning for simultaneous spinning of poly(lactic acid) and poly(vinyl acetate) to prepare two separated thermal transitions for the SMP composite. Consequently, the SMP fibers presented a triple shape memory property. Additionally, they incorporated graphene nanoplatelets into the composite to enhance the triple SME [145].

In some studies, postmodifications were applied to modify the functionality of SMP fibers, such as enhancing the mechanical properties and electrical conductivity [16,140]. Therefore, both the fillers and postmodification process such as coating provide the fibers with improved properties.

### 7.4. Electrospinning along with UV Irradiation

UV irradiation of photo cross-linkable polymers is a polymerization method that forms semicrystalline reversible phase and chemically cross-linked net-points. The obtained polymers show an exceptional thermal-induced SME with shape fixity and shape recovery higher than 90% [146]. Photocuring can be utilized before, after, or simultaneously with the electrospinning process. In Yao et al.’s work, the photo prepolymerization method was employed to make a cross-linked polymer, which improved the stability of polymer network and induced the SME in the final electrospun membrane [147].

**Figure 5:**
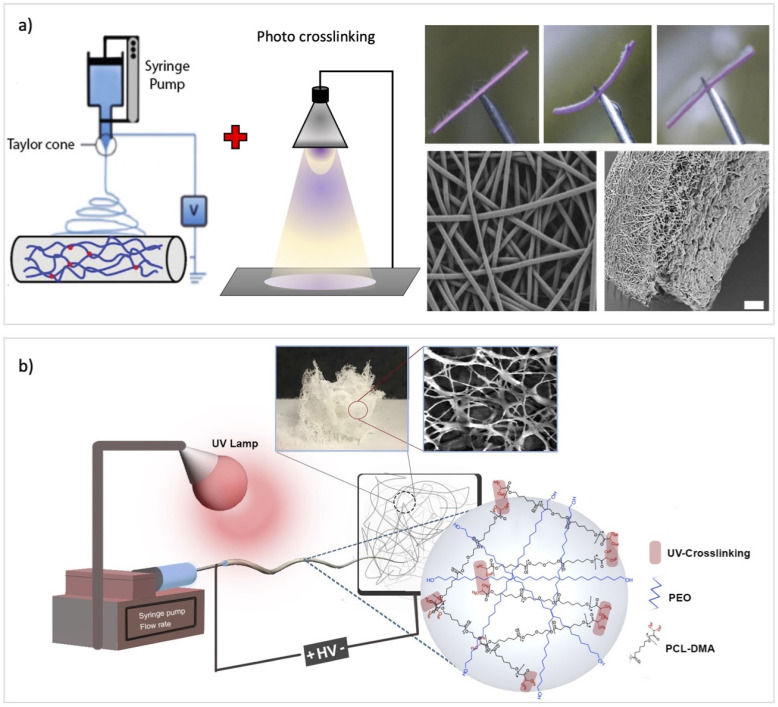
(**a**) Conventional electrospinning method with subsequent UV crosslinking (with permission from Wiley, Copyright 2015 [95]); (**b**) electrospinning and simultaneous photocrosslinking (Reprinted with permission from ref. [55]. Copyright 2019 Elsevier).

Researchers also used electrospinning with subsequent UV curing to achieve a SMP fibrous scaffold. As an example, Iregui et al. obtained a fiber-based structure with SME using electrospinning of a blend of diglycidyl ether of bisphenol-A (DGEBA) and PCL followed by UV radiation. The construct demonstrated shape memory properties over several cycles, with shape fixity ratios and shape recovery ranging between 95–99% and 88 and 100%, respectively [17]. Zhang et al. also presented a three-step fabrication process consisting of electrospinning, photocrosslinking, and programming to produce a reversible PCL-based fibrous SMP [148]. In another study, Jiang et al. used two photocrosslinkable polymers a non-stimuli-responsive thermoplastic polyurethane and a thermoresponsive copolymer of N-isopropyl acrylamide to produce a highly porous bilayer nanofibrous mat as a superfast actuator with large scale movements (Figure 5a). They observed a very fast actuation rate (less than a second) for the produced constructs and claimed that their method can be generally used for the fabrication of self-folding bilayer 3D structures suitable for 3D bioscaffolds and superfast actuators [95].

In some manufacturing procedures, UV irradiation has been applied in parallel with the electrospinning process to crosslink the polymer network for the production of shape memory fibers [32,149]. Zare et al. used this process to fabricate a 3D porous structure via crosslinking PCL-dimethacrylate within electrospun fibers (Figure 5b) [55].

Table 1 summarizes the recent studies on the fabrication of SMP micro-/nanofibrous structures using electrospinning processes.

## 8. Biomedical Applications for SMP Fibers

Electrospun SMP structures combine the shape memory property with fiber features such as large specific surface area, high porosity, and permeability. Polymer chains usually have higher mobility and therefore a lower *T*_trans_ and enhanced SME within electrospun fibers compared to the polymer films due to lower constraints-imposed neighbor chains [13,150]. Additionally, SMP fibers exhibit a faster shape recovery rate compared to the films during the same heating/cooling process, corresponding to the large specific surface area and porous structure of fibers [14]. Such properties make a suitable candidate for tissue engineering (such as bioscaffolds and cell supports). Furthermore, SMP fibers with specific structures such as core-shell structures have a great potential to be used as drug carriers and artificial blood vessels [16,17,18,19].

Shape memory porous scaffolds responding to temperature, water, magnetic field, NIR irradiation, and ultrasound stimuli have superior properties for biomedical applications. Among them, thermoresponsive SMP scaffolds with a transition temperature ~36–38 °C have gained more attention as they can easily transform to their original shape around body temperature.

SMP micro-/nanofibrous structure have extensively been used as bone tissue engineering scaffolds for treating bone defects by providing an appropriate substrate for cell growth [151]. The ideal bone scaffold should be designed to precisely occupy bone defects with irregular geometries. As SMP scaffolds can be designed to deform as desired, and then recover their original shape after stimulation, these scaffolds are suitable candidates for filling irregular bone defects via minimally invasive surgery. Furthermore, therapeutic drugs and growth factors can be encapsulated within such SMP scaffolds and released over an extended period. Liu et al. [104] fabricated a growth factor encapsulating SMP scaffold which deformed into a temporary architecture and subsequently recovered its original shape after implantation in the body. Inorganic nanoparticles, such as calcium phosphates and HAps, can also be utilized in the form of composites with SMPs or in the coatings of SMP fibers to fabricate SMP bone scaffolds [116,117].

**Figure 6:**
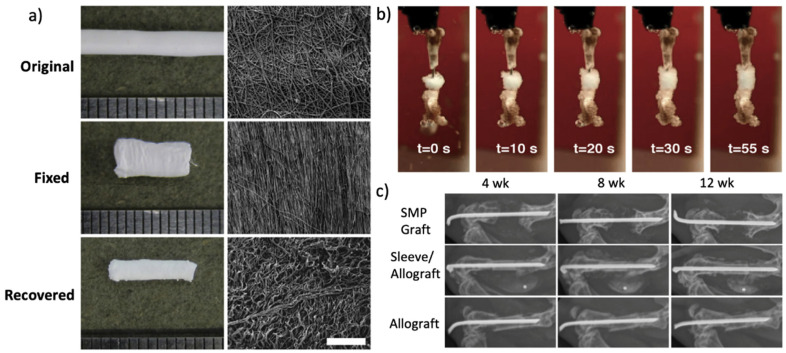
(**a**) Electrospun fibers were randomly oriented before fixing, aligned along the circumference, and randomly oriented again after recovery; (**b**) the SMP graft filled the femoral defect in 45 °C water; (**c**) radiographs of mice treated at 4, 8, and 12 weeks post-surgery (Reprinted with permission from ref. [153]. Copyright 2016 Elsevier).

Biodegradable and biocompatible SMP fibers of poly (d,l-lactide-co-trimethylene carbonate) were fabricated by electrospinning for bone tissue engineering [106]. The researchers investigated the morphology of osteoblasts on these electrospun nanofibrous scaffolds to verify the application of these scaffolds in healing various bone defects and in bone regeneration. Zare et al. also synthesized and characterized a PCL based SMP and fabricated 3D sponge-like scaffold using simultaneous electrospinning and photocrosslinking as bone tissue engineering scaffolds [55]. As another example, Torbati et al. prepared light emitting SMP fibers of poly (vinyl acetate) shrunk upon heating or immersing in water [152]. Baker et al. introduced a SMP fiber graft for the stabilization of segmental defects where the self-deploying graft could expand and contract during the surgical operation in a mouse segmental defect model in vivo. After 12 weeks, the graft could consequently integrate with the native bone and improve the defect stability (Figure 6) [153].

**Figure 7:**
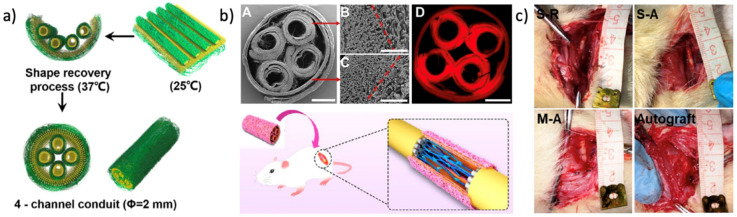
(**a**) Self-forming of the nerve conduit based on a shape memory nanofibrous scaffold; (**b**) the cross-section im-ages of the SMP multichannel nerve conduit (**A**–**C** are SEM images showing the aligned and random fiber layers, and **D** is fluorescence micrograph of the conduit); (**c**) photographs of nerve conduit transplantations at 12 weeks of postsurgery (Reprinted with permission from ref. [154]. Copyright 2020 American Chemical Society).

In a recent study, Wang et al. constructed a self-forming multichannel nerve conduit based on a degradable SMP fibrous scaffold. The electrospun SMP nanofibers mat was initially prepared in a plane form suitable for cell loading and then triggered by a physical temperature to transform to its final tubular form automatically to make a multichannel conduit (Figure 7). The promising results of the cell proliferation and repair of rat sciatic nerve defects evidenced the considerable potential of smart fibers in peripheral nerve regeneration [154].

Many micro-/nanostructures developed as ECM replacements are almost static (although they degrade over time) and thus, fail to match the dynamic physiological conditions in vivo. Notably, SMPs can endow the bioscaffolds with dynamic shifting functions to regulate cell behaviors and promoting tissue growth. Recent works on SMP fibrous structures for biomedical applications have been summarized according to the chemical compositions, fabrication techniques, actuation methods, and their biomedical applications in Table 1. This summary proves the growing interest over time in the electrospinning of SMPs with various developed electrospinning techniques for tissue engineering, drug delivery, actuators, wound healing, and cell regulation. Due to the importance of regulating cell behavior, further details have been separately discussed in the following section.

## 9. Regulation of Cell Behaviors Using Biomimetic SMP Nanofibers

Biological cells can respond to the dynamically changing ECM microenvironment, which is called topological perception [184]. This capability of cells can affect their morphology, proliferation, differentiation, migration, and gene expression [185]. Therefore, substrates with nanotopography similar to the natural ECM may influence cell behaviors and accordingly, cell adhesion and cell alignment [186]. Typically, the topography of the substrates is static and passive, unable to mimic the dynamic geometry of the cell culture microenvironments [184]. Meanwhile, dynamic structures can switch their surface geometric properties under stimulation and further affect the interactions with the cells.

**Figure 8:**
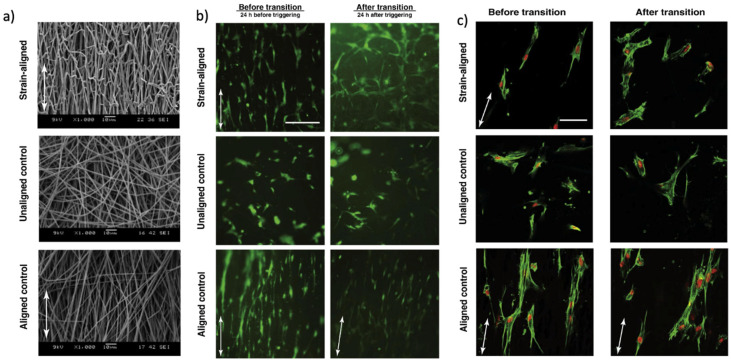
(**a**) The SEM images show the fiber alignment in the direction of applied strain following programming; (**b**) high cell viability before and after changes in the scaffold architecture; and (**c**) cell morphology and nuclear alignment changes after scaffold fiber alignment change (Reprinted with permission from ref. [33]. Copyright 2013 Elsevier).

The dynamic changes of SMP architecture can simulate the ECM and subsequently affect the cells’ alignment and motility. Tseng et al. [33] evaluated the SME on the stem cells’ alignment where the cells were initially cultured on a fibrous SMP scaffold at 30 °C. At this temperature, the fiber arrangement remained unchanged. By heating to body temperature, the SMP transition led to the shape change of the scaffold and the fibers alignment. While the cells were aligned well along the direction of the stretched fiber before the transition, as shown in Figure 8, after the transition, the cells randomly grew in different directions.

Zhao et al. produced SMP electrospun nanofibers based on PCL/gelatin methacryloyl (GelMA) [166] that could transform from a temporary planar shape to a tubular conformation upon increasing the temperature to ~37 °C. Human umbilical vein endothelial cells (HUVECs) cultured on the planar scaffold were spread on the inner surface of the tubular-shaped scaffold and formed a 3D cellular structure. Their results showed that the use of PCL/GelMA-based SMP for 3D cell culture could considerably improve the attachment, migration, and spreading of cells and the subsequent endothelialization, where confocal images confirmed the formation of a tubular alignment of HUVECs after the shape transition of SMP scaffolds. The regulation of cell behaviors using biomimetic SMP nanofibers have been reported by several research groups, as summarized in Table 2.

The dynamic behavior of SMP scaffolds may also alter cells via imposing different mechanical forces, including mechanical stresses and contractile tensions. Although cells can respond to the mechanical stresses, these forces can affect the biological signals, morphology, and functions of the cells [187]. Therefore, the effects of the residual stresses released during the shape transition of SMPs on the regulation of cell behaviors and tissue growth still have to be further evaluated in vitro and in vivo.

## 10. Conclusions and Future Challenges

In the present review, recent progress in the architecture, triggering methods, and biomedical applications of SMP micro-/nanofibers fabricated by the electrospinning process were summarized. The biomimetic SMPs with dynamical fibrous structures suitable for regulating cell behaviors, drug delivery, bone repair, scaffolding, and wound healing were described. These SMP scaffolds can be implanted via minimally invasive surgery to fill nonuniform defects.

Despite considerable progress in the synthesis and fabrication of SMPs, several challenges still remain to be addressed in the future.

(i) Two-way or reversible SMPs have greater potential compared to one-way SMPs. The evaluation of two-way reversible SMPs in the form of micro-/nanostructures for biomedical fields should be investigated.

(ii) The shape recovery rate of most SMP micro-/nanofibers is uncontrollable when responding to an external stimulus. Thus, it is necessary to optimize parameters controlling the dynamic changes of the constructs particularly for the regulation of cell behaviors.

(iii) Although many research papers have evaluated the cell behaviors after culturing on SMP fibrous scaffolds to analyze the dynamic changes of fibers in vitro, it is still a challenge to evaluate the effects of the recovery process and degradation rate on cells in vivo, especially for bone repair and skin tissue regeneration.

(iv) Due to the wide applications of thermoresponsive SMP fibers, the chemical compositions of such SMP must be optimized to achieve the accurate transition temperatures based on particular applications.

(v) Additionally, manufacturing technologies are necessary to be developed for making unique structures and properties of SMPs. Developments in manufacturing technologies, notably a combination of electrospinning and 3D printing technology simplifies the SMP production and enables the fabrication of complicated fibrous constructs using appropriate SMPs.

Overall, micro-/nano-SMPs structures have shown great potentials to serve biomedical applications. However, their wide-spread clinical applications require extensive preclinical investigations intertwined with advances of high-precision fabrication technologies and material chemistry.

## Figures and Tables

**Table 1:** A summary of recent works on SMP fibrous structures for biomedical applications.

SMP Components	Stimulation	Fabrication Method	Application	Research Team
Poly (ε-caprolactone) Polydimethylsiloxane	37 °C	Simple electrospinning	Medical shrinkable tubing and wire	Hsieh et al., 2020 [155]
Poly (lactide-co-trimethylene carbonate)	37 °C	Simple electrospinning	Peripheral Nerve Repair	Wang et al., 2020 [154]
Poly (3-Hydroxybutyrate-co-3-Hydroxyvalerate) Modified Poly(l-Lactide)	37 °C	Simple electrospinning	Bone tissue engineering	Wang et al., 2020 [156]
D,l-lactide-co-glycolide diol poly (ε-caprolactone) diols poly-l-lactide diol	42 °C	Simple electrospinning	Drug delivery	Bil et al., 2020 [157]
Poly (ε-caprolactone) Hydroxyapatite	37–45 °C	Simple electrospinning	Drug carrier	Lv et al., 2020 [117]
Polyurethane Hydroxyapatite	50 °C	Simple electrospinning	Tissue engineering	Nahavandizadeh et al., 2020 [116]
Polyurethane	65 °C	Simple electrospinning	Actuator	Guan et al., 2020 [158,159]
Poly (lactide–glycolide–trimethylene carbonate)	37 °C	Simple electrospinning	Regulating cell behavior	Chen et al., 2019 [160]
Polylactic acid Cellulose nanocrystals	57.1 °C	Simple electrospinning	Biology basic membranes	Peng et al., 2019 [119]
Poly (ε-caprolactone) Epoxy	42 °C	Electrospinning + UV irradiation	Sensors and membranes	Iregui et al., 2019 [161]
Poly (ε-caprolactone) Polyethylene oxide	39 °C	Electrospinning + UV irradiation	Tissue engineering	Zare et al., 2019 [55]
Poly (ε-caprolactone)	37 °C	Simple electrospinning	Regulating cell behavior	Niiyama et al., 2019 [162]
Poly (lactic acid)	40 °C	Simple electrospinning	Tissue engineering	Leones et al., 2019 [163]
Poly (lactic acid) Poly (vinyl acetate)	38–41 °C	Dual electrospinning	Bone tissue engineering	Sabzi et al., 2019 [164]
Poly (*ε*-caprolactone) Clay montmorillonite Epoxy	40 °C	Simple electrospinning	Tissue engineering	Dong et al., 2018 [165]
Poly (*ε*-caprolactone) Gelatin methacrylate	37 °C	Simple electrospinning	Vascular grafts	Zhao et al., 2018 [166]
Poly (lactic acid)	70 °C	Simple electrospinning	Sensors and actuators	Zhang et al., 2018 [140]
Poly (lactic acid)	Electricity	Coaxial electrospinning	Actuator	Zhang et al., 2018 [140]
Poly (ethylene glycol) Poly (ε-caprolactone) Poly (dimethylsiloxane)	Water & Heat 35 °C	Simple electrospinning	Tissue engineering	Ang et al., 2017 [139]
Poly (ε-caprolactone)	55 °C	Simple electrospinning	Sensors and actuators	Pandini et al., 2017 [54]
Poly (lactic acid)	37 °C	Simple electrospinning	Regulating cell behavior	Wang et al., 2017 [137]
Poly (ε-caprolactone) Poly (ethylene glycol)	37 °C	Simple electrospinning	Bone graft substitutes	Baker et al., 2016 [153]
Poly (lactide-trimethylene carbonate) Hydroxyapatite	43.5 °C	Coaxial electrospinning	Bone tissue engineering	Bao et al., 2016 [107]
Poly (vinyl alcohol) Polyether block amide Elastomer	85 °C	Simple electrospinning	Sensors and actuators	Shirole et al., 2016 [167]
Ethylene glycol Ethylene oxide Polypropylene oxide	38.06 °C	Simple electrospinning	Tissue engineering	Budun et al., 2016 [168]
Triethoxysilane-terminated poly (ε-caprolactone)	37 °C	Electrospinning + sol–gel	Tissue engineering	Merlettini et al., 2016 [169]
Polydimethylsiloxane Poly (ε-caprolactone)	38 °C	Simple electrospinning	Nerve tissue engineering	Dan et al., 2016 [170]
Poly (ε-caprolactone) Polyethylene oxide	55 °C	Simple electrospinning	Tissue engineering	Yao et al., 2015 [171]
Poly (*N*-isopropylacrylamide)	35 °C	Simple electrospinning	Actuator	Jiang et al., 2015 [95]
Poly (ε-caprolactone) Epoxy	63.8 °C	Simple electrospinning	Self-healing capability	Yao et al., 2015 [172]
Poly (ε-caprolactone) diol Graphene oxide	37.48 °C	Simple electrospinning	Wound healing	Tan et al., 2015 [10]
Co-polyetherester-urethane	40–45 °C	Coaxial electrospinning	Tissue engineering	Zhang et al., 2015 [143]
Poly (ε-caprolactone) Epoxy	42.3 °C	coaxial electrospinning	Tissue engineering	Zhang et al., 2015 [126]
Poly (vinyl acetate) Poly (ε-caprolactone)	16 and 55 °C	Dual electrospinning	Sensors and actuators	Birjandi et al., 2015 [173]
Poly (ε-caprolactone) Graphene	50 °C	Simple electrospinning	Sensors and actuators	Yoo et al., 2014 [138]
Poly (lactide trimethylene carbonate)	39.7 °C	Coaxial electrospinning	Drug delivery	Xianliu et al., 2014 [174]
Poly (vinyl acetate)	50 °C	Simple electrospinning	Sensors and actuators	Torbati et al., 2014 [153]
Poly (lactide-trimethylene carbonate)	39 °C	Simple electrospinning	Bone tissue engineering	Bao et al., 2014 [106]
Polyacrylonitrile (PAN)	Electricity	Simple electrospinning	Tissue engineering	Zhang et al., 2014 [175]
Poly (ε-caprolactone) Polyethylene oxide	Water	Simple electrospinning	Water responsive actuator	Gu et al., 2013 [176]
Poly (ε-caprolactone) diol	36.5 °C	Simple electrospinning	Electroactive application	Rana et al., 2013 [177]
POSS polylactide/caprolactone copolymer	40 °C	Simple electrospinning	Regulating Cell behavior	Tseng et al., 2013 [33]
Epoxy Poly (ε-caprolactone)	30 and 60 °C	Simple electrospinning	Sensors and actuators	Fejos et al., 2013 [178]
Lignin	Moisture	Simple electrospinning	Actuator	Dallmeyer et al., 2013 [91]
Poly (ε-caprolactone) multiwalled carbon nanotubes Fe_3_O_4_	40 °C Magnetic field	Simple electrospinning	Tissue engineering	Gong et al., 2012 [32]
Poly (ω-pentadecalactone) Poly (ε-caprolactone)	53 °C	Simple electrospinning	Tissue engineering	Matsumoto et al., 2012 [136]
Poly (ε-caprolactone) diol	45.5–47.5 °C	Simple electrospinning	Tissue engineering	Chen et al., 2012 [13]
Poly (ε-caprolactone) diol	38 °C	Simple electrospinning	Intelligent clothing	Chung et al., 2011 [109]
Poly (p-dioxanone) Poly (ε-caprolactone)	32–35 °C	Simple electrospinning	Tissue engineering	Kratz et al., 2011 [179]
Poly (ε-caprolactone)	50.5 °C	Simple electrospinning	Actuator	Zhang et al., 2011 [14]
Poly ferrocenyl methyl vinyl silane	Electricity	Simple electrospinning	Electric Actuator	McDowell et al., 2010 [180]
4-vinyl- benzyl chloride glycidyl methacrylate	UV irradiation	Simple electrospinning	Smart drug delivery	Fu et al., 2009 [181]
Poly (ε-caprolactone) diol	50 °C	Melt spinning	Sensors and actuators	Meng et al., 2008 [182]
Polyester polyol-based polyurethane	55 °C	Melt spinning	Sensors and actuators	Kaursoin et al., 2007 [124]
Poly (ε-caprolactone) diol	36.20 °C	Wet spinning	Tissue engineering	Meng et al., 2007 [183]

**Table 2:** A summary of recent studies on the regulation of cell behaviors using biomimetic SMP nanofibers.

Research Group	Micro-/Nanofibrous SMP	Analyses of Cell Behavior
Chen et al., 2019 [160]	Poly(lactide–glycolide)/chitosan	Regulating cell adhesion, proliferation, and morphology
Niiyama et al., 2019 [162]	Poly(ε-caprolactone) with hexamethylene diisocyanate/1,4-butanediol	Altering human mesenchymal stem cell alignment and orientation
Tseng et al., 2013 [33]	POSS containing polylactide/caprolactone copolymer	Controlling cell alignment and morphology
Wang et al., 2020 [156]	Poly(3-Hydroxybutyrate-co-3-Hydroxyvalerate) Modified Poly(l-Lactide)	Enhanced osteogenesis-inducing ability in bone mesenchymal stem cells
Wang et al., 2018 [188]	Poly(D,L-lactic acid-co-trimethyl carbonate	Providing the necessary support and guidance for motor neuron differentiation Improving the viability of embryonic stem cells and their differentiation toward motor neurons
Zhao et al., 2018 [166]	Poly-ε-caprolactone and gelatin methacrylate	Supporting homogeneous endothelial cell attachment Offering a visible approach for facile 3D endothelialization
Wang et al., 2017 [137]	Poly-DL-lactic acid-based polyurethane	On-command guidance of polarized cell motility and alignment
